# An Organic Solvent-Free Method for the Extraction of Ellagic Acid Compounds from Raspberry Wine Pomace with Assistance of Sodium Bicarbonate

**DOI:** 10.3390/molecules27072145

**Published:** 2022-03-26

**Authors:** Ning Jin, Shouyu Zhang, Shibo Sun, Minghuo Wu, Xiaojing Yang, Jianqiang Xu, Kun Ma, Shui Guan, Weiping Xu

**Affiliations:** 1 School of Ocean Science and Technology & Panjin Institute of Industrial Technology, Dalian University of Technology, Panjin 124221, China; jinningdut@163.com (N.J.); zshouyu@mail.dlut.edu.cn (S.Z.); wumh@dlut.edu.cn (M.W.); yangxiaojing@dlut.edu.cn (X.Y.); jianqiang.xu@dlut.edu.cn (J.X.); 2 School of Life and Pharmaceutical Sciences, Dalian University of Technology, Panjin 124221, China; sunshibo@mail.dlut.edu.cn (S.S.); makunonline@dlut.edu.cn (K.M.); 3 State Key Laboratory of Fine Chemicals, Dalian R&D Center for Stem Cell and Tissue Engineering, School of Chemical Engineering, Dalian University of Technology, Dalian 116023, China; guanshui@dlut.edu.cn; 4 Key Laboratory of Industrial Ecology and Environmental Engineering, Dalian University of Technology, Dalian 116024, China

**Keywords:** ellagic acid, extraction, raspberry, wine pomace, sodium bicarbonate, response surface methodology

## Abstract

Industrial processing of raspberry juice and wine generates considerable byproducts of raspberry pomace. Ellagic acids/ellagitannins, being characterized by their antioxidant and antiproliferation properties, constitute the majority of polyphenolics in the pomace and are valuable for recovery. In the present study, we developed a novel procedure with sodium bicarbonate assisted extraction (SBAE) to recover ellagic acid from raspberry wine pomace. Key parameters in the procedure, i.e., sodium bicarbonate concentration, temperature, time and solid/liquid (S/L) ratio, were investigated by single factor analysis and optimized subsequently by Response Surface Methodology (RSM). Optimal parameters for the SBAE method here were found to be 1.2% (*w*/*v*) NaHCO_3_, 1:93 (*w*/*v*) S/L ratio, 22 min and 100 °C. Under these conditions, the ellagic acid yield was 6.30 ± 0.92 mg/g pomace with an antioxidant activity of 79.0 ± 0.96 μmol Trolox eq/g pomace (DPPH assay), which are 2.37 and 1.32 times the values obtained by extraction with methanol–acetone–water solvent, respectively. The considerable improvement in ellagic acid extraction efficiency could be highly attributed to the reactions of lipid saponification and ellagitannin hydrolysis resulted from sodium bicarbonates. The present study has established an organic solvent-free method for the extraction of ellagic acid from raspberry wine pomace, which is feasible and practical in nutraceutical applications.

## 1. Introduction

The raspberry (*Rubus idaeus* L.) fruit is a well-known red juicy berry, belonging to the *Rosaceae* family and distinguishing itself by high ellagitannin contents compared to other plants in the same family, e.g., blackberry and strawberry [1,2]. The potential health benefits of raspberry and raspberry-based products are greatly attributable to polyphenolic compounds they contain, including ellagitannins, anthocyanins, flavanols, and flavonols [3,4]. A recent study has investigated the polyphenolics in the raspberry and found that ellagitannins account for 64.2% of all the polyphenolics; on the other hand, transforming raspberry into juice would only bring 32% of total ellagitannins into the juice, and the remaining ellagitannins are retained in the press cake [4]. Since global raspberry production has increased from 875,898 tons in 2018 to 895,771 tons in 2020 (https://www.fao.org/faostat/en/#data/QCL/visualize, accessed on 2 February 2022), and industrial processing of raspberries into juice or wine generates 10–12% (*w*/*w*) pomace as byproduct [5], it would be valuable to recover the ellagitannin compounds from these byproducts and turn them into food ingredients or dietary supplements.

**Figure 1:**
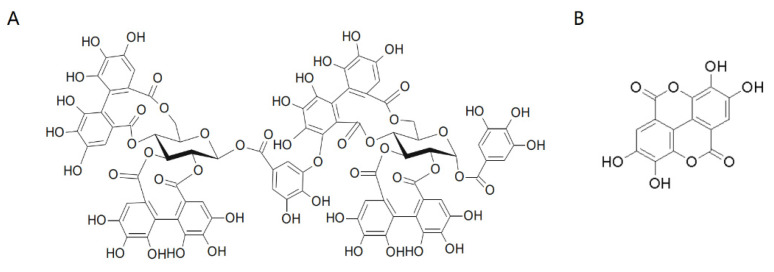
The chemical structure of sanguiin H-6 (**A**) and ellagic acid (**B**).

The majority of ellagitannins reported in the raspberry fruit are the trimer and dimer of ellagic acid, i.e., lambertianin C and sanguiin H-6, while free ellagic acid constitutes a minor proportion in the total ellagic acid pool (Figure 1) [6,7]. However, metabolism studies have shown that ellagitannins with different polymerization degrees are hydrolyzed into single ellagic acid molecules and further metabolized in the small intestine and colonies [1,8,9]. From the nutritional perspective, ellagitannins have been found to exhibit strong antioxidant activities [10,11,12,13,14,15,16], and anti-inflammatory effects [3,17,18,19,20], with great capability to weaken gastric ulcers, inhibit the growth of bacteria [21,22], fungi [23] and virus [24,25] and suppress the proliferation of cervical cancer cells [26,27]. In addition, ellagic acid has also received much attention for its antioxidant [28], anti-inflammation [29], and antiviral activities [30]. Moreover, it plays a protective role in our body against different cancer and tumor types, including colon [31], lung and esophagus cancer [32], as well as mammary tumor [33]. Thus, both oligomeric ellagitannins and free ellagic acid are good nutraceuticals, which are valuable for extraction from the raspberry pomace.

The procedures to extract ellagitannins/ellagic acid from raspberry pomace usually involve treatment with a solvent followed by High Performance Liquid Chromatography (HPLC) to determine the compound concentrations. The solvent used for extraction could be generally allocated into three categories: neutral organic [2,4,34], acidic organic [35,36,37] and basic organic [5,38]. Additionally, the procedure is usually associated with thermal, ultrasonic or microwave treatments [39,40,41]. Methanol and acetone were the most common solvent for extraction, and 4 M HCl or 4 M NaOH were widely selected for ellagitannin hydrolysis [35,38]. Extraction with organic solvent could be time-consuming and less environmentally friendly, not to mention the fact that the side effect of organic contamination in the final product. Consequently, a more environmentally friendly, time- and cost-efficient method should be developed for this application. A recent study integrated thermostable alkaline protease treatment to extract polyphenols from raspberry pomace and received satisfactory results with a 48% yield improvement compared to the conventional methanol–acetone–water approach [5]. In our experiments, raspberry wine pomace derived from wine making was used as the raw material for extraction. Several extraction reagents were compared and it was found that sodium bicarbonate could be another effective reagent to extract ellagic acid, as it is cost-efficient and eco-friendly; however, further optimization of the extraction method was required.

In this study, a novel method to extract ellagic acid from raspberry wine pomace with sodium bicarbonate was developed. The key processing parameters, i.e., sodium bicarbonate concentration, temperature, extraction time and solid/liquid (S/L) ratio, were firstly investigated with single factor analysis, followed by subsequent optimization by Response Surface Methodology (RSM). The optimized conditions were further validated by examining the ellagic acid extraction rate and its antioxidant activity. Finally, potential mechanisms of sodium bicarbonate assisted extraction (SBAE) were discussed.

## 2. Results

### 2.1. Extraction Results with Organic Solvents

In our study, 2.66 ± 0.35 mg/g ellagic acid was extracted from the milled raspberry pomace by methanol–acetone–water (MAW) method, while 1.98 ± 0.12 mg/g ellagic acid was obtained by acetone–acetic acid–water (AAW) method. This difference could be partially explained by the S/L ratio difference, which was in total 1:45 for MAW and 1:20 for AAW.

### 2.2. Extraction Results with Food Additive Solutions

**Figure 2:**
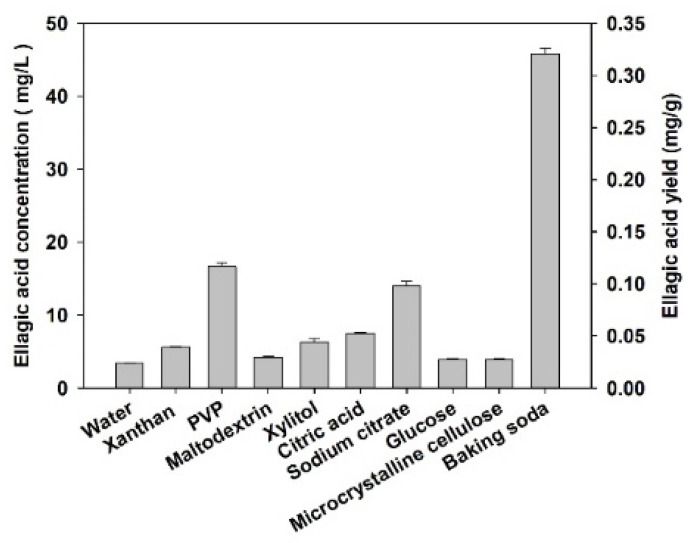
Ellagic acid extraction with different food additive solutions. Data are illustrated as mean + standard deviation (*n* = 3).

Except for the baking soda, the ellagic acid concentration extracted by the other food additive solutions ranged from 3.43–16.7 mg/L, and the yield varied between 0.024 and 0.117 mg/g, while the baking soda reagent achieved 45.8 ± 0.8 mg/L concentration and 0.321 ± 0.006 mg/g yield (Figure 2). As ellagic acid extracted by baking soda could result in a significantly higher yield compared to the other food additives (*p* < 0.001), the extraction method assisted with analytical sodium bicarbonate was further studied.

### 2.3. Single Factor Assay

**Figure 3:**
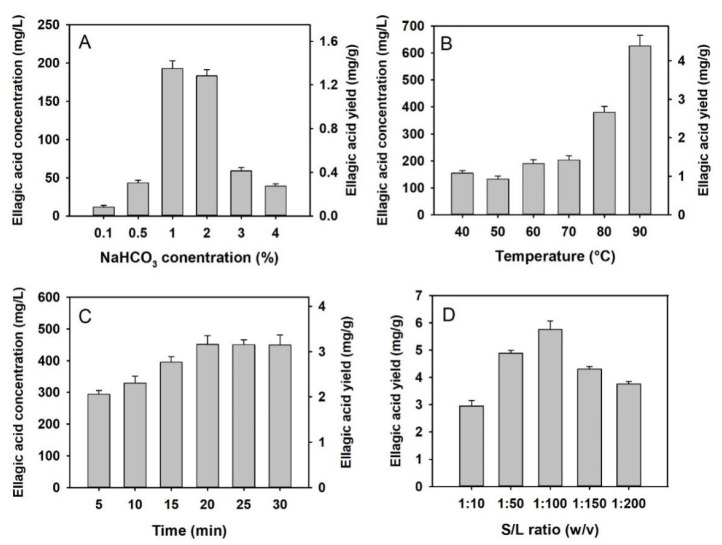
Single factor assays of the extraction method with sodium bicarbonate assistance. (**A**) factor of NaHCO_3_ concentration; (**B**) factor of extraction temperature; (**C**) factor of extraction time; (**D**) factor of S/L ratio. Data are represented as mean + standard deviation (*n* = 3).

The concentration and yield of ellagic acid were significantly affected by the factors of NaHCO_3_ concentration, temperature, time and solid/liquid (S/L) ratio (Figure 3). When extracted at 60 °C for 1 h, the ellagic acid yields from 1% and 2% NaHCO_3_ solution were significantly higher than that of 0.1%, 0.5%, 3% and 4% NaHCO_3_ solution, being of 1.35 ± 0.07 mg/g and 1.28 ± 0.06 mg/g, respectively (Figure 3A). Temperature revealed a significant positive correlation with the ellagic acid yield, and when extracted at 90 °C with 1% NaHCO_3_ for 1 h, the ellagic acid yield reached 4.38 ± 0.28 mg/g (Figure 3B). As for the extraction time, the ellagic acid yields showed similar positive correlation as the duration lengthened from 5–20 min, but stayed at 3.14–3.16 mg/g for duration times over 20 min at 100 °C and 1% NaHCO_3_ (Figure 3C). Finally, an S/L ratio of 1:100 resulted in a significantly higher yield than other S/L ratios, as the result was 5.76 ± 0.30 mg/g at 100 °C, 1% NaHCO_3_ and 20 min (Figure 3D). Therefore, the optimal parameter ranges for the SBAE approach were: the NaHCO_3_ concentration of 0.5–2%, the temperature of 100 °C, the extraction time of 10–30 min and the S/L ratio of 1:50–1:150, which were all used as the inputs for subsequent RSM design.

### 2.4. Response Surface Analysis

Based on the BBD design of response surface analysis, 17 extraction attempts were carried out, and the ellagic acid yields were illustrated in Table 1.

**Figure 4:**
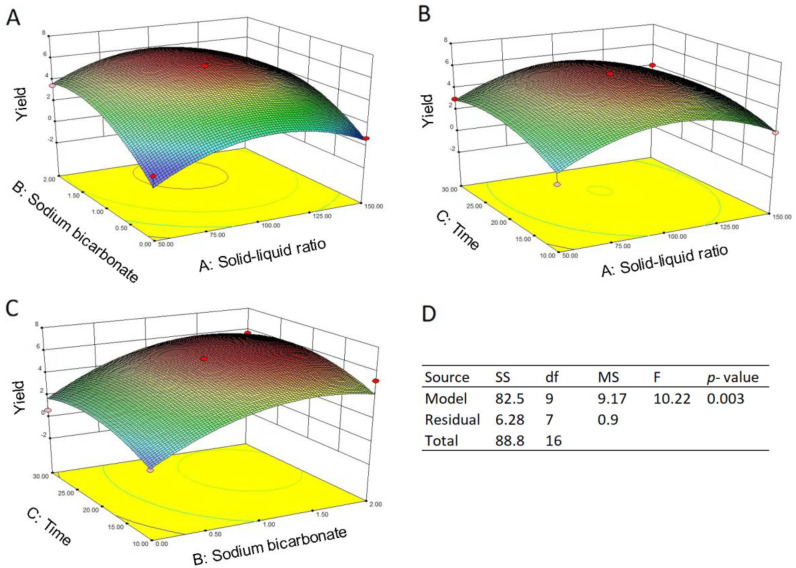
Response surface plots and ANOVA table for the quadratic model. (**A**–**C**) response surface plots refer to the NaHCO_3_ concentration, S/L ratio, time and their mutual effects on the extraction yield of ellagic acid; (**D**) ANOVA results for the quadratic model.

The response surface plots about the effects of NaHCO_3_ concentration, extraction time, S/L ratio and their mutual interaction on the ellagic acid yield were shown in Figure 4. Generally, a continual distribution for ellagic acid yield was observed in each of the three response surface plots (Figure 3A–C), and the highest response value was revealed at approximately 1% NaHCO_3_, an S/L ratio of 1:100 (*w*/*v*) and a time of 20 min. Regression and ANOVA analysis demonstrated that the relationship between the ellagic acid yield and extraction parameters is quadratic with good regression coefficient (R^2^ = 0.9293). The model significance was confirmed by an F value of 10.22 and a *p* value of 0.003 (Figure 3D), and the quadratic polynomial regression equation was concluded as the following Equation (1):

(1) $$y=6.10+0.26X_{1}+1.82X_{2}+0.63X_{3}-0.34X_{1}X_{2}-0.13X_{1}X_{3}+0.11X_{2}X_{3}-2.49X_{1}^{2}-1.85X_{2}^{2}-1.22X_{3}^{2}$$

where y is ellagic acid yield, *X*_1_ is S/L ratio, *X*_2_ is NaHCO_3_ concentration, and *X*_3_ is time. Based on the *F* test, *X*_2_ and *X*_1_^2^, *X*_2_^2^, and *X*_3_^2^ were the significant parameters (*p* < 0.05), but *X*_1_, *X*_3_, *X*_1_*X*_2_, *X*_1_*X*_3_, *X*_2_*X*_3_ were not (*p* > 0.05), suggesting NaHCO_3_ concentration plays a more important role than the other two parameters for extraction efficiency. Judging from the quadratic polynomial model, the optimal parameters for SBAE were 1.2% NaHCO_3_, an S/L ratio of 1:93 and an extraction time of 22 min with the predicted ellagic acid yield to be 6.277 mg/g. Extraction experiments were performed in triplicates using the parameters above, and the resulting ellagic acid yield was determined as 6.295 ± 0.917 mg/g, revealing that there was no significant difference compared to the prediction.

### 2.5. Antioxidant Activity

The antioxidant activities of SBAE extracts under optimal conditions, as well as those obtained by MAW and AAW, were measured by DPPH (1,1-diphenyl-2-picrylhydrazyl) and FRAP (ferric reducing antioxidant power) methods (Table 2). DPPH scavenging capacity for the three extracts demonstrated that the SBAE extract obtained the highest antioxidant activity than the MAW and AAW extracts (*p* < 0.001), which was consistent with the yield difference. However, antioxidant activity derived from the FRAP test showed the MAW extracts to be the highest, with the AAW and SBAE being similar (*p* = 0.003). One of the possible reasons for the low FRAP activity in SBAE extracts could be that the FRAP test required an acidic environment, which may interact with the NaHCO_3_ reagent contained in the SBAE extracts, causing a relatively low result. However, a DPPH activity of 79.0 ± 0.96 μmol Trolox eq/g and a FRAP activity of 0.631 ± 0.075 mmol Fe(II)S eq/g were still comparable to those in other studies that conducted organic solvent extraction from raspberry seeds [36], which suggests that the present SBAE extracts are of good antioxidant activity and are feasible for food and nutraceutical applications.

### 2.6. Quantificaiton of Ellagic Acid with HPLC Analysis

**Figure 5:**
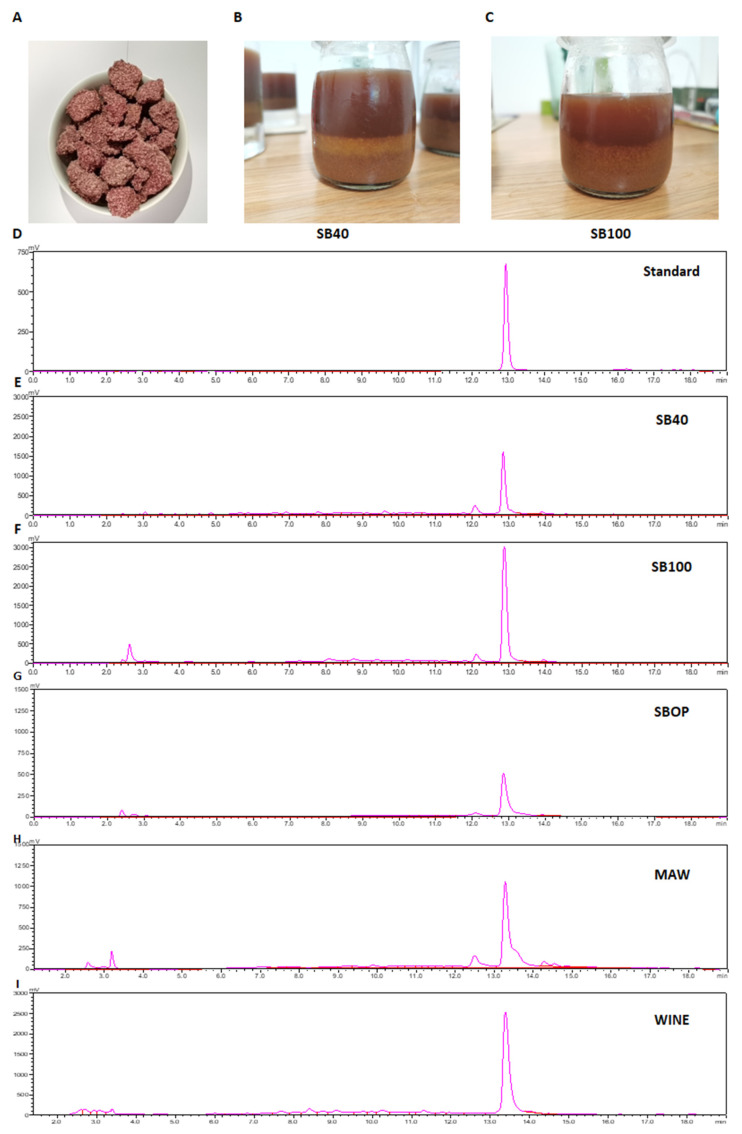
The pomace sample, extraction solution and the HPLC analysis. (**A**) raspberry wine pomace; (**B**) the extraction solution under the condition of 1% NaHCO_3_, 40 °C, 1 h, and S/L ratio of 1:10 (SB40); (**C**) the extraction solution under the condition of 1% NaHCO_3_, 100 °C, 10 min, and S/L ratio of 1:10 (SB100); (**D**) the standard of ellagic acid; (**E**) the SB40 sample; (**F**) the SB100 sample; (**G**) the optimal condition of SBAE method (SBOP), which is 1.2% NaHCO_3_, 100 °C, 22 min, and S/L ratio of 1:93; (**H**) the MAW sample; (**I**) the raspberry wine sample.

The raspberry wine pomace in this study was a dark pink color and was rich in raspberry seeds (Figure 5A). The SBAE products showed dark red to brown color, and a yellow emulsion layer was typically observed in the sample processed by low temperature, such as 40 °C (Figure 5B,C). The HPLC profiles of some typical samples were illustrated in Figure 5D–I. The retention time of ellagic acid standard was 12.94 min (λ = 254 nm), which was applied to identify ellagic acid components in the extracts. The extracts were quantified based on the integral area of HPLC curves. In all the SBAE and organic solvent extracts, as well as the raspberry wine, ellagic acid was found to be the principal polyphenolic compound (Figure 5E–I), while another type of small molecule was occasionally observed at the retention time of 2.63 min, which was further identified as gallic acid, due to its similar retention time to the gallic acid standard.

## 3. Discussion

### 3.1. The Extraction of Organic Solvents and Food Additives

Several studies have conducted ellagic acid or ellagitannin extraction with organic solvent from the whole fruit or seeds. For instance, Agnieszka et al. [2] achieved 2.9 mg/g of ellagitannin concentration from frozen fruit by 80% acetone extraction together with ultrasonic treatments; Vrhovsek et al. [6] obtained 0.97 mg/g of ellagic acid from fresh fruit by 70% acetone with 4 M HCL and an extraction temperature of 85 °C; Saad et al. [5] retrieved 10.6 mg/g total ellagic acid (ellagic acid/ellagitannins) from pomace press-cake with MAW method; and Sojka et al. [4] obtained 0.26–0.36 mg/g of free ellagic acid and 17.7–32.1 mg/g of total ellagic acid from the raspberry seed fraction using AAW method. The present study achieved a higher recovery rate for free ellagic acid than that reported previously; however, for the yield of total ellagic acid, ellagic acid conjugates and ellagitannins, the present study only achieved an intermediate level. This result could be partially explained by the different cultivars, locations, extraction solvents and materials engaged. Furthermore, it also implied the possibility that the wine pomace in this study contains high content of free ellagic acid, which may be derived from microbial fermentation during the wine making.

Although food additive solutions have been rarely used in polyphenolic extraction, they were tested in the present study due to the following two reasons: (1) to minimize the application of organic solvents, and (2) to improve the solubility of ellagic acid in the extraction solution. Generally, maltodextrin, xylitol, and glucose were selected as they are potentially capable to change water polarity; xanthan, PVP and microcrystalline cellulose were chosen because of their long chain molecular structure; citric acid, sodium citrate and baking soda were selected since they are either acidic or basic, all of which may improve the solubility of ellagic acid in water. However, the results showed no significant improvement for the solubility of ellagic acid in the extraction solution, except for baking soda, therefore the extraction method with the sodium bicarbonate assistance was further investigated.

### 3.2. Ellagic Acid Composition of the Raspberry Wine Pomace

The SBAE extract with optimal parameters of RSM assay achieved an ellagic acid yield of 6.30 ± 0.92 mg/g from the raspberry wine pomace with an antioxidant activity of 79.0 ± 0.96 μmol Trolox eq/g pomace (DPPH assay). To our knowledge, this is the highest free ellagic acid yield as a result of extracts from raspberry-based materials so far. In previous studies, Vrhovsek et al. [6] has reported an extraction yield of 0.97 ± 0.02 mg/g for free ellagic acid from fresh raspberry fruit with acetone and 4 M HCl; Wang et al. [36] has extracted 2.14 ± 0.04 mg/g of free ellagic acid from raspberry seed fraction by acid treatment with diethyl ether/ethyl acetate solvent; and Sojka et al. [4] has achieved 0.24–0.36 mg/g of free ellagic acid from the raspberry press cakes with acetone as the solvent. A better yield of free ellagic acid in this study could be attributed to three major effects: (1) ellagic acid composition of the raspberry wine pomace; (2) saponification derived from the sodium bicarbonate; and (3) alkaline hydrolysis induced by sodium bicarbonate thermal treatment.

The present study utilized raspberry wine pomace as the raw material for extraction, which is unprecedented to our knowledge. To profile the composition of ellagic acid pool, i.e., free ellagic acid, ellagic acid-conjugates and ellagitannins, it would be better to profile the extracted compounds with HPLC and consider the extracts from extraction methods with organic solvents of low acid/alkaline strength, since highly acidic or basic environments, especially when combined with elevated temperatures, could easily induce ellagitannins to hydrolyze [36,42]. Using 70% acetone as the extraction solvent, Sojka et al. [5] has proved that free ellagic acid constituted only 1–2% of the total ellagic acid pool, while ellagitannins constituted up to 99% of that in the press-cake pomace. Using 80% acetone associated with ultrasonication, Agnieszka et al. [2] found that the total ellagic acid pool of frozen raspberry is predominantly oligomeric ellagitannins, i.e., Sanguiin H-6 and Lambertianin C, while free ellagic acid constitutes less than 10%. However, the present study has found that principal components in the organic extract of wine pomace (Figure 5H) as well as raspberry wine (Figure 5I) were free ellagic acid instead of ellagitannins, suggesting that the fermentation of raspberry wine may greatly facilitate the transformation from ellagitannins to free ellagic acid. Therefore, the wine pomace in the present study contained higher content of free ellagic acid than juice pomace press-cakes [42], which contributed to the high yield of free ellagic acid in SBAE extracts. Since free ellagic acid molecules are more easily absorbed and metabolized by the small intestine [9], our results demonstrate that this raw material is of good value for nutraceutical application.

### 3.3. Saponification by the Sodium Bicarbonate

Since ellagic acid and llagitannins compounds are actually embedded in a complex matrix of insoluble lipids, fibers and proteins within the seeds [4,5], it would be reasonable and practical to improve the ellagic acid extraction efficiency by hydrolyzing the matrix components. The SBAE method in this study utilized NaHCO_3_ as the principal functional chemical, which may induce saponification of raspberry lipids and therefore improve the extraction performance. Clearly, saponification is greatly correlated with the temperature [43]. As shown by Figure 5B,C, SB40 product exhibited a clear yellow emulsion layer between the aqueous and solid phases, which was rather thin or barely seen in the SB100 product or other SBAE products at 100 °C. The yellow emulsion layer could contain semi-saponified raspberry lipids. High temperatures facilitated the NaHCO_3_-induced saponification [43], further improving the release of embedded ellagic acid molecules, which agreed with the findings that the ellagic acid yield was positively correlated with the extraction temperature (Figure 3B).

Similar tendencies have also been observed in studies concerning alkaline or alkaline enzyme extraction. In a recent study, Ayoub et al. [38] reported that total phenolic yield by extracting black raspberry press-cakes elevated from 0.8 to 1.9 mg GAE/g pomace with an additional 4 M NaOH. Lipid hydrolysis resulting from NaOH treatment was found to be the main factor causing the better outcome. In another study of treating raspberry press-cakes with thermostable alkaline protease [5], the extraction efficiency of total polyphenols increased from 25 to 37 mg/g, and that of total ellagic acid raised from 10.6 to 14.6 mg/g compared to organic solvent-only results, and simultaneous extraction of lipophilic and polyphenol compounds induced by alkaline protease was noted as the primary reason behind such improvements. Interestingly, both studies found oligomeric ellagitannins as the major and ellagic acid as the minor component in the ellagic acid pool, suggesting the coexistence of ellagitannins/ellagic acid associated with the lipophilic compounds in the raspberry pomace. In another earlier study, Landbo and Meyer [44] also found that by increasing pH value from neutral to slightly alkaline, the simultaneous extraction of oil and total polyphenols, including ellagitannins, was promoted. In accordance with previous findings, the enhanced extraction efficiency by the SBAE method could be partially derived from the NaHCO_3_-induced saponification during the extraction process. Therefore, temperature and NaHCO_3_ concentration were of great importance, since they affect the lipid saponification process considerably.

### 3.4. Alkaline Hydrolysis Induced by the Sodium Bicarbonate Solvent

With its optimal conditions, the ellagic acid yield obtained from SBAE elevated from 2.66 ± 0.35 mg/g to 6.30 ± 0.92 mg/g comparing to that retrieved by the organic solvent alone. In other words, a 137% improvement in the ellagic acid yield was achieved with SBAE. Other than lipid saponification, the hydrolysis of ellagitannin compounds in the alkaline thermal condition could be another reason for such finding. Sojka et al. [42] tested the stability and transformation of raspberry ellagitannins in aqueous solutions, and revealed that oligomeric ellagitannins of lambertianin C and sanguiin H-6 rapidly hydrolyzes in neutral and mildly basic media at 60–80 °C, producing ellagic and gallic acids as the main end products. Thus, it is highly possible that ellagitannin hydrolysis was promoted under the optimal SBAE parameters, which was supported by the appearance of single gallic acid molecules in the SB100 sample (Figure 5F). Similar trends have also been found in the alkaline organic [38] and alkaline protease extraction studies [5] that alkaline conditions facilitated ellagitannin hydrolysis. On the other hand, overdosing NaHCO_3_ might cause degradation of ellagic acid, which was suggested by the reduction in ellagic acid yield from 1–2% NaHCO_3_ treatment to that of 3–4% NaHCO_3_ (Figure 3A). Therefore, 1.2% NaHCO_3_ solvent derived from RSM assay might be the optimal NaHCO_3_ concentration that allows the ellagitannin hydrolysis, ellagic acid degradation and lipid saponification to be balanced.

Organic solvent extraction associated with various acidity and alkalinity levels have been widely applied to extract bioactive compounds in raspberry. For instance, Wang et al. [36] used acid hydrolysis (pH2 adjusted with HCL) associated with diethyl ether/ethyl acetate solvent to extract phenolics from raspberry seeds; Aybastier et al. [37] applied conditions of 64% methanol, 0.45 M HCl, 68 °C and 117 min to extract antioxidant compounds from blackberry leaves; and Vehovsek et al. [35] suggested 70% acetone with 4 M HCl, 85 °C and 6 h to extract ellagitannin from the raspberry fruit. The acidified solvents seem more promising due to their advantages for the denaturation of plant cell walls, the acid hydrolysis of *Rubus* polyphenols and the stabilization of extracted products [35,36,37]. However, recent practices engaging alkaline organic or alkaline protease treatment have revealed the feasibility of extraction processes in basic conditions. The SBAE method in this study utilized NaHCO_3_ (1.2% *w*/*v*, i.e., 0.14 M) as the main alkaline reagent, which is less strong than that used in previous reports, and no organic solvent or protein enzyme is required, which further controls contamination by organic reagent and cut down the cost on reagents. Under the optimal conditions of 100 °C, 1.2% NaHCO_3_, 22 min and an S/L ratio of 1:93, an ellagic acid yield of 6.30 ± 0.92 mg/g was achieved from the raspberry wine pomace with an antioxidant activity of 79.0 ± 0.96 μmol Trolox eq/g (DPPH assay), both comparable to those in previous researches [5,36,38]. These results suggest our SBAE protocol is an efficient, eco-friendly and cost-efficient method to extract ellagic acid from the raspberry wine pomace, and might prove useful in food and nutraceutical applications.

## 4. Materials and Methods

### 4.1. Sample Collection and Preparations

Raspberry wine pomace and wine samples were kindly offered by Liaoning Huayuan Winery Company (Panjin, Liaoning, China). The pomace was collected after wine making (≈1 month), dried with outdoor air for 5 days and kept indoors at room temperature for 6 months. The material was a dry solid and a dark pink color, mainly consisting of raspberry seeds. Prior to the extraction, the pomace was ball-ground into a fine powder with a SCIENTZ-48 High-throughput Tissue Grinder (Ningbo, Zhejiang, China) for 5 min at 50 Hz, and the fine powder was then stored at −20 °C before it was taken out for experiments.

### 4.2. Reagents

Acetone, methanol and acetic acid solvents were all of analytical grade and purchased from Shanghai Aladdin Bio-Chem Technology Co., Ltd. (Shanghai, China). Methanol and formic acid used for HPLC were of chromatographic grade and obtained from Spectrum Chemical Manufacturing Corp-China (Shanghai, China). Chemical reagents of ellagic acid, gallic acid, 1,1-diphenyl-2-picrylhydrazyl (DPPH), Trolox, 2,4,6-tripyridin-2-yl-1,3,5-triazine (TPTZ), FeCl_3_, FeSO_4_ and NaHCO_3_ were of analytical grade and all bought from Shanghai Aladdin. Food additives of xanthan, polyvinyl pyrrolidone (PVP), maltodextrin, xylitol, citric acid, sodium citrate, glucose, microcrystalline cellulose and baking soda (sodium bicarbonate) were of food grade with >98% purity and purchased from Youbaojia Food Additive Co., Ltd. (Weifang, Shandong, China).

### 4.3. Extraction with Organic Solvents

Ground raspberry pomace samples were treated with organic solvents by procedures in two previous reports. Firstly, ellagic acid were extracted with methanol–acetone–water (7:7:6, *v*/*v*/*v*) method (MAW) as described by Ayoub et al. [38]. Briefly, samples were mixed with methanol–acetone–water (7:7:6, *v*/*v*/*v*) solvent with a solid/liquid (S/L) ratio of 1:15 and then ultrasonicated at 30 °C for 20 min. The solution containing the extracted materials was obtained after centrifugation and the residues were subject to two more extractions in accordance with the first one. Three solutions obtained were joined together and studied for ellagic acid concentration with HPLC. Another set of ellagic acid was extracted by acetone–acetic acid–water (70:1:29, *v*/*v*/*v*) method (AAW) following the procedure described by Sojka et al. [4]. Briefly, samples were mixed with 70% acetone containing 1% acetic acid in an S/L ratio of 1:8, and subsequently ultrasonicated for 5 min and left in the dark for 15 min. After extraction, the solution was obtained after centrifugation and the residues underwent additional two extractions with an S/L ratio of 1:6, but other parameters remained the same. The three extracts were combined and looked into ellagic acid concentration by HPLC. Each procedure was performed in triplicates, and 1 g of milled raspberry pomace was used as the material for each extraction attempt.

### 4.4. Extraction with Food Additive Solutions

The food additive solutions were prepared by mixing 2 g of xanthan, PVP, maltodextrin, xylitol, citric acid, sodium citrate, glucose, microcrystalline cellulose, and baking soda with 100 mL of double distilled water, respectively. Ellagic acid was extracted by mixing the milled raspberry pomace with all the food additive solutions mentioned above in an S/L ratio of 1:10 and keeping the mixture at 60 °C for 1 h with a shaking rate of 200 rpm. The extracts were obtained after centrifugation at 8000× *g* for 5 min, and ellagic acid concentration was determined in the supernatant by HPLC. Each procedure was conducted in triplicates, and 1 g of milled raspberry pomace was used as the material for each extraction attempt.

### 4.5. Single Factor Assay

As baking soda solution revealed high performance, as deduced by the extraction assay above, single factor assay was carried out for sodium bicarbonate assisted extraction with a focus on four extraction parameters: NaHCO_3_ concentration, temperature, time and S/L ratio.

(1) For the NaHCO_3_ concentration assay, 1 g of milled raspberry pomace was extracted by 0.1%, 0.5%, 1%, 2%, 3% NaHCO_3_ water solution, respectively, in an S/L ratio of 1:10 at 60 °C for 1 h shaking (200 rpm).
(2) For the temperature assay, 1 g of milled raspberry pomace was extracted at 40 °C, 50 °C, 60 °C, 70 °C, 80 °C, and 90 °C, respectively, with 1% NaHCO_3_ solution in an S/L ratio of 1:10 for 1 h shaking (200 rpm).
(3) For the time assay, 1 g of milled raspberry pomace was extracted for 5, 10, 15, 20, 25 and 30 min, respectively, with 1% NaHCO_3_ solution at 100 °C in an S/L ratio of 1:10 S/L ratio.
(4) For the S/L ratio assay, 1 g of milled raspberry pomace was extracted in S/L ratios of 1:10, 1:50, 1:100, 1:150, and 1:200, respectively, with 1% NaHCO_3_ solution at 100 °C for 20 min.

For each assay, extraction was conducted in triplicates, and the mixture after extraction was centrifuged at 8000× *g* for 5 min, followed by the supernatant undergoing subsequent ellagic acid quantification by HPLC analysis.

### 4.6. Response Surface Methodology Assay

The RSM assay was performed with the assistance of Design Expert Software (version 8.0.6, Stat-Ease, Inc., Minneapolis, MN, USA). Three parameters, namely NaHCO_3_ concentration, time and S/L ratio, were recognized as key extraction factors and selected as the independent variables (X_1_, X_2_, and X_3_) in the RSM assay, while an extraction temperature of 100 °C was set as a constant since it demonstrated optimal outcome compared to other temperatures in the above assay. A three-variable, three-level RSM assay (NaHCO_3_ concentration, 0%, 1%, 2%; time, 10, 20, 30 min; and S/L ratio, 1:50, 1:100, 1:150, *w*/*v*) was designed by a Box–Behnken design, which required 17 extraction experiments as illustrated in Table 1. Ellagic acid yield in each experiment was determined and regarded as the response variable (y). Data were analyzed by regression analysis and response surface plots. Second-order polynomial Equation (2) was used to calculate the predicted response:

(2) $$y=b_{0}+\overset{3}{\underset{i=1}{\sum }}b_{i}X_{i}+\overset{3}{\underset{i=1}{\sum }}b_{ii}X_{i}^{2}+\overset{2}{\underset{i=1}{\sum }}\overset{3}{\underset{j=i+1}{\sum }}b_{ij}X_{i}X_{j}$$

where y is the response, *b*_0_ is the offset term, *b_i_* is the linear effect, *b_ii_* is the squared effect, *b_ij_* is the interaction effect and *X_i_* and *X_j_* are independent variables. Model adequacy was evaluated by an F test and R^2^ calculations. The optimal parameters predicted by the model were thereafter performed in triplicates, and the actual data obtained were then compared to the predictions.

### 4.7. Analysis of Antioxidant Activity

Ellagic acid extracts obtained by MAW and AAW methods, as well as those retrieved with optimal parameters in the SBAE approach, were looked into their antioxidant activity by a DPPH method described by Brand-Williams et al. [45], and a ferric reducing antioxidant power (FRAP) method reported by Benzie [46]. A calibration curve was established using Trolox or FeSO_4_ as the standard. The DPPH radical scavenging activity was expressed as micromoles of Trolox equivalents per gram of milled raspberry pomace (μmol Trolox eq/g). The FRAP activity was expressed as millimoles of ferrous sulfate equivalents per gram of milled raspberry pomace (mmol Fe(II)S eq/g). Each assay was carried out in triplicates.

### 4.8. HPLC Analysis of Ellagic Acid Content

HPLC analysis were carried out using a Shimadzu LC-16 system (Shimadzu Corporation, Suzhou, China), which consists of binary pumps, a UV detector, a ZORBAX SB-C18 (4.6 × 250 mm, 5 μm) column (Agilent Technologies Inc., Waldbronn, Germany) and a LabSolutions Essentia control system (version 5.88, Shimadzu, Kyoto, Japan). The flow rate was 1 mL/min and the injection volume was 20 μL. The monitoring wavelength for ellagic acid was 254 nm, and the mobile phase consisted of 0.3% formic acid in water (solvent A) and pure methanol (solvent B). HPLC separation was carried out by the following gradient conditions: 0–4 min 20–40% B, 4–13 min 40–90% B, 13–14 min 90–20% B, 14–16 min 20% B, with a total run time of 16 min. A standard curve to quantify ellagic acid was constructed in the range of 10, 25, 50, 100, and 200 mg/L. Sample peaks were identified based on their retention time compared with the ellagic acid standard.

### 4.9. Statistical Analysis

Data were statistically examined by one-way analysis of variance (ANOVA) with R software (version 4.0.3, R core team) [47]. Results were presented as mean ± standard deviation. Significant difference was defined as *p* < 0.05.

## 5. Conclusions

A novel method named as SBAE was developed for ellagic acid extraction from the raspberry wine pomace using sodium bicarbonate as the main functional reagent. Extraction temperature, NaHCO_3_ concentration, time and S/L ratio were the key parameters for SBAE. By an RSM assay, the optimal SBAE conditions were established with the ellagic acid yield being 6.30 ± 0.92 mg/g and the antioxidant activity being of 79.0 ± 0.96 μmol Trolox eq/g (DPPH assay). The present SBAE method requires neither organic solvent nor protease or strong acid/alkaline reagents, whereas it achieved a higher extraction rate of ellagic acid than the conventional methods. The raspberry wine pomace was rich in free ellagic acid. The NaHCO_3_-induced lipid saponification and alkaline hydrolysis of ellagitannins could be the main reasons behind the improvement of ellagic acid extraction efficiency. Feasible procedure, good yield and reliable antioxidant activity of the extracts suggest the SBAE method to be useful for the application of ellagic acid extraction in the food and nutraceutical industry.

## Figures and Tables

**Table 1:** The BBD design and results of response surface analysis.

No.	X_1_ (S/L Ratio)	X_2_ (NaHCO_3_)	X_3_ (Time)	Yield (mg/g)
1	1 (1:150)	1 (2%)	0 (20 min)	2.513
2	0 (1:100)	−1 (0%)	1 (30 min)	0.634
3	0 (1:100)	0 (1%)	0 (20 min)	6.155
4	0 (1:100)	0 (1%)	0 (20 min)	6.181
5	0 (1:100)	0 (1%)	0 (20 min)	6.207
6	1 (1:150)	−1 (0%)	0 (20 min)	0.721
7	1 (1:150)	0 (1%)	−1 (10 min)	2.032
8	−1 (1:150)	−1 (0%)	0 (20 min)	0.318
9	0 (1:100)	0 (1%)	0 (20 min)	6.187
10	−1 (1: 50)	0 (1%)	1 (30 min)	3.014
11	0 (1:100)	1 (2%)	1 (30 min)	5.659
12	1 (1:150)	0 (1%)	1 (30 min)	4.061
13	0 (1:100)	−1 (0%)	−1 (10 min)	0.634
14	−1 (1: 50)	0 (1%)	−1 (10 min)	0.454
15	−1 (1: 50)	1 (2%)	0 (20 min)	3.479
16	0 (1:100)	0 (1%)	0 (20 min)	5.760
17	0 (1:100)	1 (2%)	−1 (10 min)	5.219

**Table 2:** Ellagic acid yield and antioxidant activity of extracts derived from organic solvent method and sodium bicarbonate assisted method.

Extraction Method ^1^	Ellagic Acid Yield (mg/g)	Antioxidant Activity ^2^	
		DPPH (μmol Trolox eq/g)	FRAP (mmol Fe(II)S eq/g)
MAW	2.66 ± 0.35 b	59.7 ± 3.02 b	1.162 ± 0.080 a
AAW	1.98 ± 0.12 c	24.1 ± 0.06 c	0.679 ± 0.032 b
SBAE	6.30 ± 0.92 a	79.0 ± 0.96 a	0.631 ± 0.075 b

^1^ MAW, methanol–acetone–water (7:7:6) method; AAW, acetone–acetic acid–water (70:1:29) method; SBAE, sodium bicarbonate assisted extraction at its optimal parameters as mentioned above. ^2^ Results are expressed as μmol or mmol eq per g milled raspberry pomace. Different letters in the same column represent significant difference.

## Data Availability

Not applicable.

## Sample Availability

Samples of the compounds are not available from the authors.
